# The role of cardiac magnetic resonance imaging in the assessment of heart failure with preserved ejection fraction

**DOI:** 10.3389/fcvm.2022.922398

**Published:** 2022-07-18

**Authors:** Clement Lau, Mohamed M. M. Elshibly, Prathap Kanagala, Jeffrey P. Khoo, Jayanth Ranjit Arnold, Sandeep Singh Hothi

**Affiliations:** ^1^Department of Cardiology, New Cross Hospital, Royal Wolverhampton NHS Trust, Wolverhampton, United Kingdom; ^2^Department of Cardiovascular Sciences, University of Leicester, Leicester, United Kingdom; ^3^Department of Cardiology, Liverpool University Hospitals NHS Foundation Trust and Liverpool Centre for Cardiovascular Science, Liverpool, United Kingdom; ^4^Institute of Cardiovascular Sciences, College of Medical and Dental Sciences, University of Birmingham, Birmingham, United Kingdom

**Keywords:** HFpEF, CMR in HFpEF, diastolic function, diastolic dysfunction, diastolic heart failure

## Abstract

Heart failure (HF) is a major cause of morbidity and mortality worldwide. Current classifications of HF categorize patients with a left ventricular ejection fraction of 50% or greater as HF with preserved ejection fraction or HFpEF. Echocardiography is the first line imaging modality in assessing diastolic function given its practicality, low cost and the utilization of Doppler imaging. However, the last decade has seen cardiac magnetic resonance (CMR) emerge as a valuable test for the sometimes challenging diagnosis of HFpEF. The unique ability of CMR for myocardial tissue characterization coupled with high resolution imaging provides additional information to echocardiography that may help in phenotyping HFpEF and provide prognostication for patients with HF. The precision and accuracy of CMR underlies its use in clinical trials for the assessment of novel and repurposed drugs in HFpEF. Importantly, CMR has powerful diagnostic utility in differentiating acquired and inherited heart muscle diseases presenting as HFpEF such as Fabry disease and amyloidosis with specific treatment options to reverse or halt disease progression. This state of the art review will outline established CMR techniques such as transmitral velocities and strain imaging of the left ventricle and left atrium in assessing diastolic function and their clinical application to HFpEF. Furthermore, it will include a discussion on novel methods and future developments such as stress CMR and MR spectroscopy to assess myocardial energetics, which show promise in unraveling the mechanisms behind HFpEF that may provide targets for much needed therapeutic interventions.

## Introduction

Heart failure (HF) is a clinical syndrome caused by abnormalities in cardiac structure and function resulting in increased intracardiac pressures and/or reduced cardiac output (1). Patients with HF frequently present with dyspnea, fatigue and fluid retention, organ dysfunction due to hypoperfusion and have a higher risk of sudden cardiac death due to ventricular arrhythmia or pump failure. Although the range of pathologies resulting in impaired cardiac function is broad, current clinical guidance on the classification and management of HF places great emphasis upon left ventricular ejection fraction (LVEF). Thus, patients with HF and LVEF of 50% or greater are commonly categorized as having HF with preserved ejection fraction (HFpEF). The prevalence of HF is estimated at 64.3 million globally (2) with more than half of these people having HFpEF (3). In this review, we discuss the utility of cardiovascular magnetic resonance imaging (CMR) for the assessment of HFpEF.

In patients presenting with HFpEF, the pathological hallmarks are abnormal left ventricular (LV) filling and increased LV end diastolic pressures (LVEDP), collectively termed diastolic dysfunction. HFpEF has diverse causes associated with a multitude of co-morbidities. Established risk factors implicated in the development of HFpEF include hypertension, type 2 diabetes (T2D), chronic kidney disease and obesity. HFpEF also appears to be more prevalent in females and presents at an older age than heart failure with reduced ejection fraction (HFrEF). Importantly, atrial fibrillation (AF) has a complex association with HFpEF (4) and AF commonly coexists with HFpEF with a reported prevalence of up to 65% in older patients (5). The underlying pathological changes are thought to be related to chronic inflammation, neurohormonal activation, changes in intracellular signaling pathways, endothelial and microvascular dysfunction and myocardial fibrosis (6).

To date, a number of diagnostic algorithms have been proposed since accurate identification of HFpEF can be problematic (7). Importantly, a wide range of HFpEF clinical phenocopies also exist, each with underlying pathophysiologically distinct etiologies including hypertrophic cardiomyopathy (HCM), ATTR cardiac amyloidosis, Fabry disease iron overload cardiomyopathy and cardiac sarcoidosis. Moreover, unique disease-targeted therapies in these conditions might reverse, halt or slow disease progression.

## Diagnosing HFpEF with CMR

**Figure 1:**
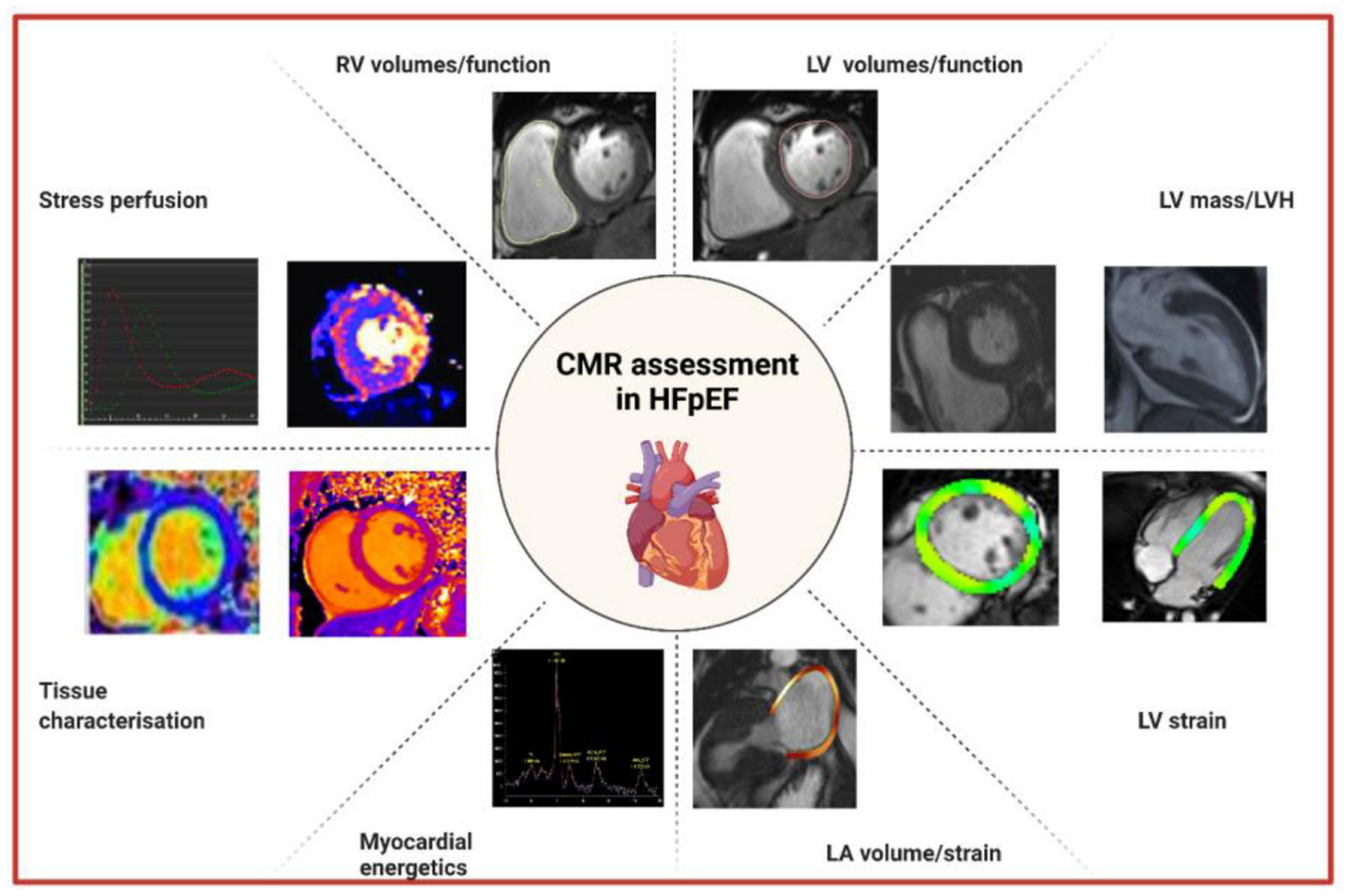
Approaches to the assessment of HFpEF by CMR. CMR, cardiovascular magnetic resonance; HFpEF, heart failure with preserved ejection fraction; LA, left atrial; LV, left ventricular; LVH, left ventricular hypertrophy; RV, right ventricular.

Cardiac imaging plays a pivotal role in the diagnosis of HFpEF. Echocardiography is the initial modality of choice to assess LV diastolic function particularly because of its availability, cost-effectiveness and technical capabilities through Doppler imaging. However, CMR is increasingly being utilized for the further evaluation of patients with HFpEF (Figure 1). CMR is currently second line to echocardiography in the imaging assessment of diastolic function as it remains a limited resource, is less economical and is challenging in patients with claustrophobia. Practical limitations in HFpEF patients include difficulties in breath holding in older patients and in obese patients. Image quality may be impacted in patients with AF and in those with cardiac devices and post valvular intervention. In addition, there remains a larger clinical and research evidence base for echocardiographic assessment of HFpEF. Despite these drawbacks, the strengths of CMR include its higher spatial resolution and ability to quantify structural changes in the heart with greater precision and reproducibility compared to other imaging modalities, and its unique tissue characterization capability (8). The diagnostic utility of CMR is highlighted by a study comparing CMR and echocardiography. Kanagala et al. (9) found that CMR diagnosed new, significant clinical cardiac pathologies such as myocardial infarction, HCM and constrictive pericarditis in 27% of 154 patients with HFpEF and these patients were at higher risk of death and HF hospitalization at 6 months. Recent data further suggests integration of different CMR measures of diastolic function into a novel diagnostic algorithm (10) in a similar vein to echocardiography but further validation and consensus agreement is required before putting such algorithms into routine clinical practice.

## LV functional assessment

CMR is the current gold standard imaging technique to assess LV volumes and therefore LVEF, and LV mass, as it avoids the geometric assumptions made by echocardiography and provides superior reproducibility (11, 12). Advances in CMR machine learning further serve to increase repeatability for measuring LV volumes and mass (13). To provide the highest spatial and temporal resolution, ECG-gated bright-blood balanced steady state free precession (SSFP) sequences of contiguous short-axis slices from the LV base to apex are acquired (14, 15). In patients with HF, cardiac remodeling and left ventricular hypertrophy (LVH) are associated with impairment of myocardial contractility and diastolic dysfunction even with preserved LV ejection fraction (16). LVH is an established risk factor for adverse cardiovascular events (17–19) and is associated with incident HF events (20).

Phase contrast CMR is routinely used to determine flow for valvular assessment using through-plane velocity encoding. These sequences can also be utilized to measure transmitral inflow, generating E (passive diastolic inflow) and A (active diastolic inflow) waves and pulmonary vein inflow, which correlate well with echocardiographic Doppler indices (21–23). However, CMR-derived values tend to be lower and may underestimate velocities compared to echocardiography, which may relate to the lower temporal resolution with CMR (30–40 ms) vs. echocardiography (<5 ms) (24, 25). Additional pitfalls of CMR include time consuming data acquisition and analysis in addition to positive or negative phase offset errors with through-plane flow imaging due to local non-compensated eddy currents (26). The recently developed CMR golden-angle method permits acquisition of 150 to 250 frames per cardiac cycle to match that of echocardiography (27). This novel method involves k-space lines acquired continuously determined by the golden-angle of each sector, together with alternating velocity-encoding signs (27). Advances in CMR sequences have led to the development of 3D and 4D flow sequences which may prove to be useful for a more advanced assessment of diastolic filling than measurement of mitral inflow and pulmonary vein inflow by phase contrast CMR alone. For example, a 3D velocity-encoded MRI with retrospective mitral valve annular plane tracking sequence had better agreement with Doppler echocardiography for LV diastolic filling patterns compared to a 2D one-directional velocity-encoded sequence (28). Furthermore, in 53 healthy volunteers, a comparison of 4D flow CMR kinetic energy to mitral inflow E/A ratio showed a stronger independent association with age than standard 2D metrics (29).

Tissue Doppler imaging (TDI) for the quantification of myocardial velocities is a fundamental, clinically validated method for the assessment of diastolic function by echocardiography (30, 31). Similarly, phase contrast CMR can be used to determine myocardial e′ velocities (peak modal velocity in early diastole by pulsed TDI waveform at the mitral valve annulus). CMR-derived mean e′ and E/e′ have consistently shown excellent correlation with echocardiographic values in patients with diastolic dysfunction (23, 32). In a small observational study of patients with hypertensive heart disease (*n* = 18), CMR E/e′ strongly correlated with invasively measured mean pulmonary capillary wedge pressure (PCWP) (*r* = 0.8, *p* < 0.0001) and had a 100% positive predictive value for E/e′ < 8 and PCWP ≤ 15 mmHg, and similarly so for E/e′ > 15 and PCWP >15 mmHg (32). Measurement of CMR LV fractional area change during the first 30% of diastole (termed diastolic-index) in the short axis view correlated well with e′ on echocardiography (33).

## LV strain

Although the use of LVEF is clinically the dominant imaging method for defining severity, subtype and progression of HF, it has limitations (34). Other imaging parameters, particularly LV strain may detect changes in myocardial architecture and function before changes in LVEF occur (35). Strain is a measure of myocardial mechanics that describes the deformation of LV myocardial fibers which are orientated in the longitudinal, circumferential and radial directions. Changes in longitudinal function occur early on in HF (36). Accordingly, global longitudinal strain (GLS) has been identified as an important marker of early myocardial dysfunction (37). Indeed, GLS has proven to have powerful prognostic value superior to LVEF (38). In a systematic review involving 5,721 patients with cardiovascular disease (CVD), there was stronger independent association with mortality with each SD change in the absolute value of baseline GLS (HR 0.50, 95% CI 0.36–0.69; *p* < 0.002) compared to LVEF (HR 0.81, 95% CI 0.72–0.92; *p* = 0.572) (38). Different methods to measure strain by CMR have evolved from myocardial tagging and phase contrast velocity-encoding to feature tracking, the latter now being the more commonly employed sequence.

Strain measurement by myocardial tagging using Spatial Modulation of Magnetization (SPAMM) involves tagging orthogonally intersecting sets of lines marking rectangular grids in a 2D image (39). In 1,500 participants from the MESA (Multi-Ethnic Study of Atherosclerosis) cohort, diastolic function from circumferential strain curves independently predicted incident HF and atrial fibrillation over an 8-year follow-up period (40). However, the requirement to acquire dedicated images and time-consuming post-processing has meant that myocardial tagging has not gained routine clinical use for the assessment of LV diastolic function. Strain-encoded (SENC) imaging uses tags parallel to the image plane rather than as a series of orthogonal lines (41). In systole, the tagged planes compress together leading to a shift in the peak spectrum location in *k* space (42). The rate of shift is then used to determine strain. Compared to conventional tagging, SENC can be acquired in half the time (43) and provides higher temporal resolution of strain measurements through the entire cardiac cycle (41). Fast-SENC techniques can shorten the image acquisition duration down to a single heartbeat (44).

An alternative method to assess myocardial deformation is by phase contrast velocity-encoding which offers better spatial resolution but lower temporal resolution than myocardial tagging (45). This allows measures of instantaneous velocity over a short time period. Displacement encoding with stimulated echoes (DENSE) is a free-breathing, phase-velocity based method which permits measurement of displacement during most but not all of the cardiac cycle (46). Unfortunately, this limits the assessment of diastolic tissue displacement and acquired images have low signal to noise ratio.

**Figure 2:**
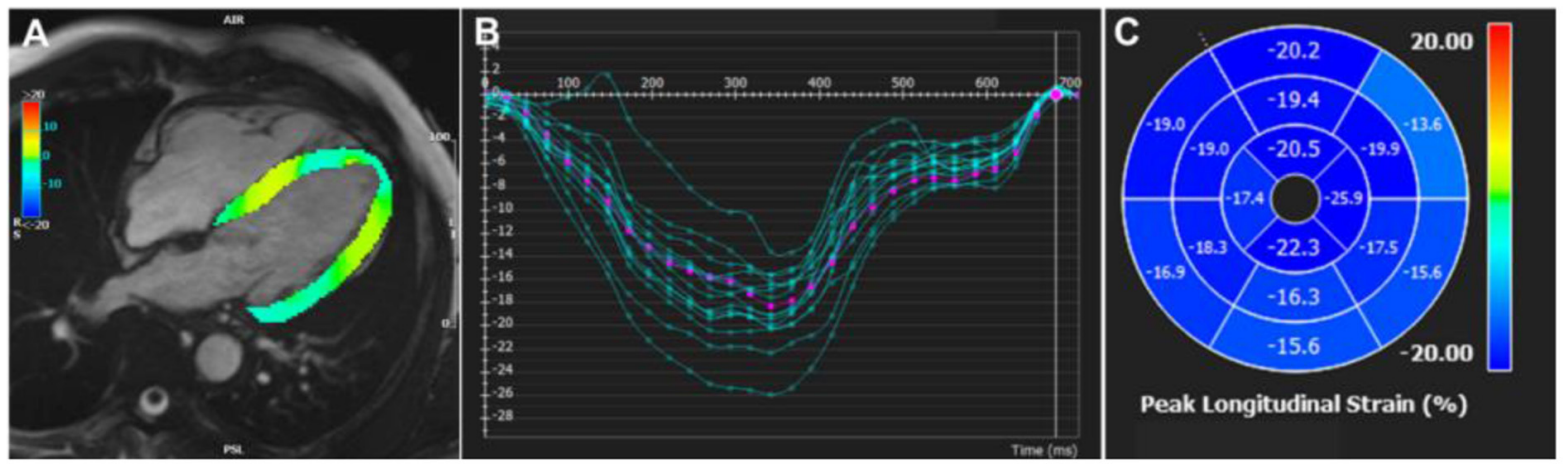
Feature tracking CMR strain analysis. **(A)** Feature tracking CMR in the 4-chamber view generating **(B)** longitudinal strain curves, **(C)** displayed as a polar map for each AHA segment. AHA, American Heart Association.

Feature tracking (FT) CMR analysis (FT-CMR) tracks points or features in the myocardium across successive imaging frames over the whole cardiac cycle, generating strain values and curves (Figure 2). FT-CMR is increasingly favored for the assessment of strain due its close correlation with echocardiographic speckle tracking (47) and myocardial tagging (48, 49). Moreover, the ability to utilize routine SSFP-based cine images simplifies clinical workflows. In a small study of 18 HFpEF patients compared to 18 age and sex-matched controls, GLS independently predicted abnormal relaxation index, Tau, by invasively measured pressure-volume loops (50). GLS measured by FT-CMR proved to be a powerful independent predictor of all-cause mortality in a multi-center study of 1,012 patients with HFrEF and a median follow-up of 4.4 years (51). In a study of 131 patients with HFpEF, GLS ≥ −8% by FT-CMR independently predicted HF hospitalization and cardiovascular death at 2.5 years follow up (52). Novel applications of FT-CMR include the assessment of LV torsion, rotation and diastolic recoil (53). Interestingly, different rotational mechanics are found in patients with amyloidosis and HCM, conditions which often masquerade as HFpEF (54).

## Left atrial size

Changes in left atrial size (LA) are a hallmark of elevated LV filling pressures in patients with HFpEF (55). Maximal LA volume measured at end-systole in sinus rhythm and indexed to body surface area (BSA) has strong predictive value for adverse cardiovascular events (56). CMR provides a more accurate measurement of LA size compared to echocardiography, owing to inherently superior spatial resolution. LA size can be measured by contouring the LA in the short axis stack or, more commonly, by the area-length method or Simpson's biplane in the 4-chamber and 2-chamber views. Although Simpson's biplane is the reference standard to assess LA size by echocardiography, there is limited consensus on the preferred method by CMR (57, 58). Nevertheless, in a multicenter study of nearly 11,000 subjects with a median follow up of 4 years, increased BSA-indexed LA size measured by CMR was independently associated with all-cause mortality (59).

## Left atrial function

LA function can be subdivided into its reservoir (LA filling during LV systole), conduit (passive LV filling in early to mid-diastole) and pumping (LA contraction to augment LV filling in late diastole) phases. LA conduit-filling capacity is calculated by maximum LA volume minus pre-atrial contraction volume whilst LA active filling capacity is calculated by pre-atrial contraction volume minus the minimum LA volume (60). LA stroke volume is defined as maximum minus minimum LA volume. Furthermore, LA ejection fraction (LAEF) can be derived from these measured volumes with both biplane and short axis methods showing good agreement in sinus rhythm (61). Left atrial ejection fraction is closely associated with LVEDP on cardiac catheterization (62).

Increased LA volumes, reduced LAEF, reduced LA reservoir and booster pump strains are all associated with diastolic dysfunction as well as its severity (63). In the multi-ethnic population-based Dallas Heart Study of 1,802 patients, lower LAEF was independently associated with increased mortality [hazard ratio per 1 SD (8.0%): 1.56 (1.32–1.87)] with superior and incremental predictive value over maximum LA volume index (64). These findings were further supported by the MESA study of 536 patients with T2D, in whom incident cardiovascular disease was strongly and independently associated with lower passive, active and total LAEF (65). Kanagala et al. demonstrated that LAEF is reduced in HFpEF (*n* = 140) compared to controls (*n* = 48) and that a lower LAEF was associated with an increased risk of all-cause mortality or first HF hospitalization (log-rank, all *p* = 0.028; sinus *p* = 0.036) (61, 66). Furthermore, the strong association with adverse outcomes was similar for LAEF derived by either the biplane or short axis methods during CMR (61).

## Left atrial strain

Application of CMR-FT to the LA generates strain data that have been evaluated as measures of LA function (67). Chirinos et al. (68) compared patients with HFpEF (*n* = 101), HFrEF (*n* = 120) and without HF (*n* = 640) demonstrating that conduit and reservoir LA strain measured using CMR-FT independently predicted risk of incident HF admission or mortality. In the MESA cohort, incident HF was predicted by lower longitudinal atrial strain (25 ± 11% vs. 38 ± 16%; *p* < 0.001) and lower LA emptying fraction (40 ± 11 vs. 48 ± 9%; *p* < 0.001) at baseline (69). In a small study of 22 HFpEF patients compared to heathy controls, LA conduit strain was significantly reduced in HFpEF and was associated with impaired oxygen uptake (VO_2_ max) during cardiopulmonary exercise testing and invasive measurements of impaired early LV filling (70).

## Right ventricle

CMR is also the gold standard non-invasive method to assess right ventricular (RV) size and function. In a prospective, observational study the prevalence of RV dysfunction (RVEF < 47%) as determined by CMR was present in 19% of individuals with HFpEF (*n* = 135) (71). Furthermore, RV dysfunction was independently associated with death and HF hospitalization (adjusted HR 3.946, 95% CI 1.878–8.290, *p* = 0.0001). (71). HFpEF is a recognized cause of elevated pulmonary artery pressures (PAP) and pulmonary hypertension. Resultant changes in the right heart readily assessed by CMR include increased right atrial size, RV hypertrophy and septal bowing. Assessment of diastolic dysfunction must take into account an estimation of PAP which is typically elevated in patients with HFpEF. Echocardiography can estimate systolic PAP by measurement of the peak tricuspid regurgitant (TR) velocity using the formula PAP = 4^*^(TRV_max_)^2^. While measurement of the tricuspid regurgitant jet peak velocity is readily assessed by echocardiography, this is less readily performed by CMR. However, systolic PAP can be indirectly estimated by identifying the peak TR velocity using phase contrast CMR flow analysis at the level of the tricuspid valve (72).

## Myocardial tissue characterization

In HFpEF, alterations in the extracellular matrix with increased collagen deposition are thought to be a result of inflammation and increased oxidative stress (73). This process leads to myocardial fibrosis, which may contribute to the impaired relaxation that is observed in HFpEF. CMR has the unique capability to detect both focal replacement myocardial fibrosis using late gadolinium enhancement (LGE) and diffuse interstitial fibrosis through parametric mapping sequences (native T1 and extra cellular volume (ECV) quantification).

The presence of replacement fibrosis by LGE in patients at risk of HFpEF including in AF (74) and diabetes (75) has been shown to increase the risk of mortality. Furthermore, identifying LGE can provide additional risk stratification for patients at risk for hospitalization for HF regardless of etiology or LV systolic dysfunction (76). In an observational cohort study of 1,096 patients with AF, LA fibrosis by LGE was associated with an overall incidence of developing HF at 3.1% per year (77). Moreover, 80% of patients developed HFpEF (*n* = 63) rather than HFrEF (*n* = 20) after a median 2.7 years follow-up and that the incidence of HF increased with increasing LA fibrosis.

T1 measures the time taken for longitudinal relaxation of excited protons to return to equilibrium following application of a radiofrequency pulse. Colored maps can be generated so that pixel values represent the T1 in each voxel. T1 values in the myocardium and blood pool acquired pre- and post-contrast, and by accounting for hematocrit, can be used to calculate ECV. ECV quantifies the relative expansion of the extracellular matrix acting as a validated surrogate imaging biomarker for myocardial fibrosis (78, 79).

**Figure 3:**
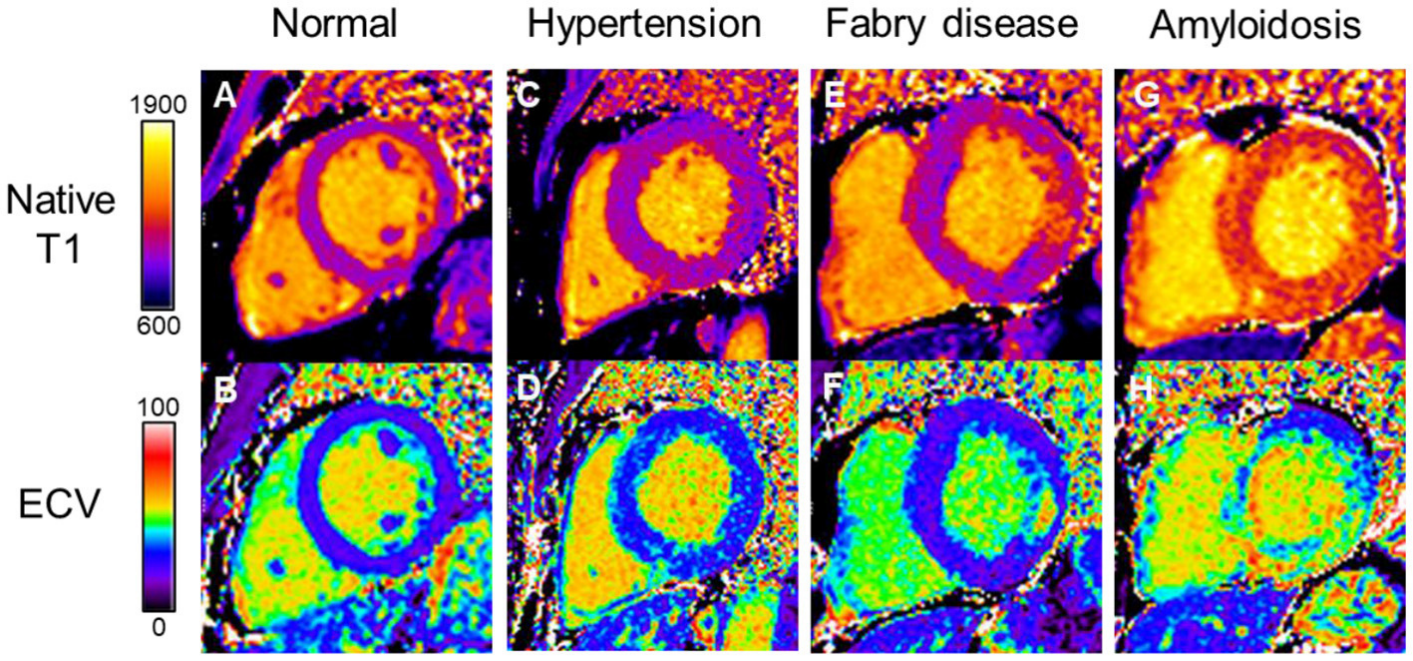
Myocardial tissue characterization by native T1 and ECV differentiating disease processes in HFpEF. **(A)** Normal native T1 and **(B)** normal ECV maps. Hypertensive heart disease with LVH showing diffuse myocardial fibrosis on **(C)** native T1 map and **(D)** increased ECV. Fabry disease showing **(E)** globally reduced native T1 values and **(F)** replacement fibrosis in the basal inferolateral wall on ECV map; Cardiac amyloidosis showing globally elevated **(G)** native T1 and **(H)** ECV values. Color scales are represented for native T1 in milliseconds and ECV in percentage. ECV, extracellular volume; HFpEF, heart failure with preserved ejection fraction; LVH, left ventricular hypertrophy.

Both native T1 mapping and ECV are helpful in detecting inherited and acquired cardiomyopathies, particularly Fabry disease and cardiac amyloidosis (Figure 3). Diffuse myocardial fibrosis by native T1 mapping and ECV appears to be associated with diastolic dysfunction and LV stiffness in HFpEF (*n* = 62) but not in HFrEF (*n* = 40) or healthy controls (*n* = 22) (80). ECV has been demonstrated to correlate with invasive measures of load-independent passive LV stiffness (81, 82). In a study comparing patients with HFpEF, hypertension and healthy controls, ECV was significantly increased in HFpEF (35.9 ± 5.0%) compared to both hypertensive patients (31.9 ± 5.2%) and healthy controls (27.0 ± 4.3%) (83). Moreover, ECV was superior to GLS in differentiating between HFpEF and hypertension.

ECV may have a unique role to play in identifying HFpEF phenotypes at higher risk of cardiovascular events and death (84). In a cohort of 410 patients at risk for or diagnosed with HFpEF, ECV correlated with BNP levels and outcomes of heart failure hospitalization or death (85). This may indicate that the degree of myocardial fibrosis occurs in a continuum of severity and may precede overt clinical features of HFpEF. These findings are corroborated by other studies demonstrating that a lower post-contrast T1 time (median <388 ms) is a strong predictor of adverse events (86). Furthermore, both focal and diffuse myocardial fibrosis was noted to be more prevalent in HFpEF (*n* = 140), compared to age and sex matched control subjects. ECV indexed to body surface area correlated with LV mass:volume ratio, RV end-diastolic volume index and maximum LA volume index and independently predicted adverse outcomes (87). Interestingly, a recent study demonstrated that anterior RV insertion point fibrosis measured by increased native T1 times significantly correlated with markers of increased LV end-diastolic pressures and filling (88).

## Stress perfusion and exercise stress CMR

Patients with HFpEF may only develop dyspnea on exertion and have limited indices of diastolic dysfunction at rest. The gold standard for diagnosing the effects of HFpEF during exertion is invasive right heart catheterization during exercise (89), although it is more often assessed indirectly by exercise stress echocardiography (90). Recent studies have shown a role for stress perfusion or CMR combined with exercise to increase the diagnostic yield for HFpEF (91–93). In particular, there appears to be significant coronary microvascular dysfunction in HFpEF patients as revealed by myocardial perfusion reserve following stress perfusion imaging (94). Stress perfusion CMR identifies patients at higher risk of major cardiovascular events in HFpEF without known coronary artery disease (95, 96).

In a feasibility study, exercise stress combined with real-time CMR was able to detect HFpEF, confirmed by right heart catheterization, with high accuracy (93). The authors also demonstrated that LA longitudinal shortening was the most accurate parameter to detect HFpEF. In future, exercise stress CMR may play a prominent role alongside exercise echocardiography in the workup of HFpEF patients, without the need for invasive tests.

## Myocardial energetics

The high energy requirement of the heart and minimal capacity to store energy suggests that an imbalance of energy supply and demand may predispose to the development of myocardial dysfunction. MR spectroscopy has the capability to assess myocardial energetics by measuring phosphocreatine to adenosine-triphosphate (PCr/ATP) ratio, and cardiac steatosis by measuring myocardial triglyceride content (MTG). In a small study of 12 patients with T2D and diastolic dysfunction, there were reductions in PCr/ATP ratio when compared to controls (97). Burrage et al. (98) investigated patients with a spectrum of diastolic dysfunction including T2D (*n* = 9), HFpEF (*n* = 14) and cardiac amyloidosis (*n* = 9) and controls (*n* = 11). Across the spectrum of HFpEF, there was a decrease in PCr/ATP ratio in parallel to increases in E/e′, NT-proBNP and lower LV diastolic filling rates with low workload exercise. Moreover, patients with HFpEF and cardiac amyloidosis had transient pulmonary congestion with exercise as revealed by pulmonary proton density mapping. In HFpEF, significantly greater MTG and therefore myocardial steatosis correlates with reductions in CMR measured diastolic strain and VO_2_max (99).

Recent clinical trials have identified beneficial effects of therapeutics on cardiac metabolism. Evidence has been growing for the sodium–glucose cotransporter 2 (SGLT2) inhibitor, empagliflozin, for the treatment of HFpEF (100). The mechanism of these benefits have not been fully elucidated but an improvement in cardiac metabolism has been shown following in a significant increase in PCr/ATP in a small study of 18 patients with T2D compared to 10 healthy volunteers (101). The authors also found an increase in mean LVEF of 7% and a 3% increase in GLS.

Epicardial adipose tissue (EAT) surrounding myocardium and within the pericardium is metabolically active providing energy to myocardium through the breakdown of triglycerides and also generates pro-inflammatory mediators (102). Furthermore, EAT may affect mechanical properties of ventricular function. In obese HFpEF (*n* = 99) compared to non-obese HFpEF (*n* = 97) and healthy controls (*n* = 71), there was significantly higher total epicardial heart volume [945 ml (831–1,105 ml) vs. 797 ml (643–979 ml) and 632 ml (517–768 ml); *p* < 0.0001] and EAT thickness (10 ± 2 vs. 7 ± 2 and 6 ± 2 mm; *p* < 0.0001) measured by CMR (103). Furthermore, the larger epicardial heart volume in obese HFpEF was associated with increased pericardial restraint and ventricular interdependence.

## Novel methods in assessing HFpEF by CMR

Recent advances in CMR have generated novel sequences and measures to assess HFpEF. Diastolic dysfunction results from altered LV compliance leading to changes in LV filling patterns. LV time-volume and peak filling rate curves generated using CMR acquired LV volumetric datasets associate with the severity of echocardiographic-derived diastolic dysfunction (104). Patients with HFpEF have a lower peak filling rate adjusted for end diastolic volume (105), prolonged peak filling rates (106) and greater diastolic volume recovery (proportion of diastole required for recovery of 80% of stroke volume) by CMR (107).

A fundamental aspect of HFpEF assessment is to determine PAP. Vortices of blood, measured by 3D or 4D phase-contrast CMR, appear in the main pulmonary artery in pulmonary hypertension (108, 109). Measurement of the duration of blood vortices allows estimation of pulmonary artery pressures (110). A vortex duration ≥15% corresponds to an invasive mean PAP ≥25 mmHg. Using 4D flow analysis there is good correlation to Doppler echocardiographic estimates with potentially higher diagnostic yields in the detection of raised pulmonary artery pressures (111).

Central transit times have long been to known to be increased in HF (112). Using first pass perfusion, Cao and colleagues (113) demonstrated that global central transit times from the right atrium to aorta were significantly prolonged in HFpEF, which correlated with increased PCWP. Measurement of central transit time may therefore act as an additional marker of HFpEF for patients undergoing CMR though data on its use remains limited.

Experimental CMR sequences in the pipeline may further serve to characterize the myocardium and alterations in diastolic function potentially adding to the diagnostic role of CMR in the assessment of HFpEF. Examples include diffusion tensor imaging (114), MR elastography (115), artificial intelligence and radiomics (116), which are beyond the scope of this review article.

## Conclusion

Assessment by CMR enables refinement of a clinical HFpEF diagnosis into underlying cardiac conditions such as HCM and cardiac amyloidosis. Furthermore, the strengths of CMR over other imaging modalities include the capability for sub-categorization into pathophysiological sub-types such as increased myocardial fibrosis, left atrial dysfunction or microvascular dysfunction. The comprehensive evaluation of HFpEF by CMR may enable risk profiling of patients with HFpEF and perhaps allow focused, targeted therapies in the future. Advances in CMR sequences and postprocessing are generating novel indices of diastolic dysfunction which not only aid diagnosis but may hold prognostic risk prediction. The role of CMR in assessment of diastolic dysfunction continues to evolve at a rapid rate but large-scale studies are still required to permit technical reproducibility as well as clinical validation across different populations and subcategories of HFpEF. Moreover, future research could focus on the challenge of identifying which established and novel indices should be routinely incorporated into clinical workflows. It remains to be determined how such advances compare to a predominant echocardiographic based approach to clinical diagnosis and management of HFpEF, especially whether the use of CMR can lead to improved prognostication and outcomes.

## Author contributions

CL drafted the manuscript, performed literature review, and created figures. ME created a figure and reviewed the manuscript. PK and JK critically appraised the manuscript and made constructive modifications and suggestions. JA codevised the topic for the manuscript, critically appraised the manuscript, and made constructive modifications and suggestions. SH devised the idea for the manuscript, led the whole project, critically appraised the manuscript from inception, and co-wrote the manuscript from the outset. All authors contributed to the article and approved the submitted version.

## Conflict of interest

The authors declare that the research was conducted in the absence of any commercial or financial relationships that could be construed as a potential conflict of interest.

## Publisher's note

All claims expressed in this article are solely those of the authors and do not necessarily represent those of their affiliated organizations, or those of the publisher, the editors and the reviewers. Any product that may be evaluated in this article, or claim that may be made by its manufacturer, is not guaranteed or endorsed by the publisher.

## References

[1] McDonagh TA Metra M Adamo M Gardner RS Baumbach A Böhm M. 2021 ESC Guidelines for the diagnosis and treatment of acute and chronic heart failure. Eur Heart J. (2021) 42:3599–726. 10.1093/eurheartj/ehab368 34447992

[2] James SL Abate D Abate KH Abay SM Abbafati C Abbasi N. Global, regional, and national incidence, prevalence, and years lived with disability for 354 diseases and injuries for 195 countries and territories, 1990–2017: a systematic analysis for the Global Burden of Disease Study 2017. Lancet. (2018) 392:1789–858. 10.1016/S0140-6736(18)32279-7 30496104 PMC6227754

[3] Lam CSP Donal E Kraigher-Krainer E Vasan RS. Epidemiology and clinical course of heart failure with preserved ejection fraction. Eur J Heart Fail. (2011) 13:18–28. 10.1093/EURJHF/HFQ121 20685685 PMC3003453

[4] Kotecha D Lam CSP Van Veldhuisen DJ Van Gelder IC Voors AA Rienstra M. Heart failure with preserved ejection fraction and atrial fibrillation: vicious twins. J Am Coll Cardiol. (2016) 68:2217–28. 10.1016/J.JACC.2016.08.048 27855811

[5] Sartipy U Dahlström U Fu M Lund LH. Atrial fibrillation in heart failure with preserved, mid-range, and reduced ejection fraction. JACC Heart Fail. (2017) 5:565–74. 10.1016/J.JCHF.2017.05.001 28711451

[6] Shah SJ Kitzman DW Borlaug BA van Heerebeek L Zile MR Kass DA. Phenotype-specific treatment of heart failure with preserved ejection fraction. Circulation. (2016) 134:73–90. 10.1161/CIRCULATIONAHA.116.021884 27358439 PMC4930115

[7] Ho JE Zern EK Wooster L Bailey CS Cunningham T Eisman AS. Differential clinical profiles, exercise responses, and outcomes associated with existing HFpEF definitions. Circulation. (2019) 140:353–65. 10.1161/CIRCULATIONAHA.118.039136 31132875 PMC6684250

[8] Arnold JR McCann GP. Cardiovascular magnetic resonance: applications and practical considerations for the general cardiologist. Heart. (2020) 106:174–81. 10.1136/HEARTJNL-2019-314856 31826937

[9] Kanagala P Cheng ASH Singh A McAdam J Marsh AM Arnold JR. Diagnostic and prognostic utility of cardiovascular magnetic resonance imaging in heart failure with preserved ejection fraction - implications for clinical trials. J Cardiovasc Magn Reson. (2018) 20:1–12. 10.1186/S12968-017-0424-9 29321034 PMC5763769

[10] Ramos JG Fyrdahl A Wieslander B Thalén S Reiter G Reiter U. Comprehensive cardiovascular magnetic resonance diastolic dysfunction grading shows very good agreement compared with echocardiography. JACC Cardiovasc Imaging. (2020) 13:2530–42. 10.1016/j.jcmg.2020.06.027 32828779

[11] Bottini PB Carr AA Prisant LM Flickinger FW Allison JD Gottdiener JS. Magnetic resonance imaging compared to echocardiography to assess left ventricular mass in the hypertensive patient. Am J Hypertens. (1995) 8:221–8. 10.1016/0895-7061(94)00178-E 7794570

[12] Myerson SG Bellenger NG Pennell DJ. Assessment of left ventricular mass by cardiovascular magnetic resonance. Hypertension. (2002) 39:750–5. 10.1161/hy0302.104674 11897757

[13] Bhuva A Bai W Lau C Davies R Ye Y Bulluck H. A multicenter, scan-rescan, human and machine learning cmr study to test generalizability and precision in imaging biomarker analysis. Circ Cardiovasc Imaging. (2019) 12:e009214. 10.1161/CIRCIMAGING.119.009214 31547689

[14] Barkhausen J Ruehm SG Goyen M Buck T Laub G Debatin JF. MR evaluation of ventricular function: true fast imaging with steady-state precession versus fast low-angle shot cine MR imaging: feasibility study. Radiology. (2001) 219:264–9. 10.1148/radiology.219.1.r01ap12264 11274568

[15] Childs H Ma L Ma M Clarke J Cocker M Green J. Comparison of long and short axis quantification of left ventricular volume parameters by cardiovascular magnetic resonance, with ex-vivo validation. J Cardiovasc Magn Reson. (2011) 13:1–9. 10.1186/1532-429X-13-40 21834992 PMC3169477

[16] Palmieri V Bella JN Dequattro V Roman MJ Hahn RT Dahlof B. Relations of diastolic left ventricular filling to systolic chamber and myocardial contractility in hypertensive patients with left ventricular hypertrophy (the PRESERVE study). Am J Cardiol. (1999) 84:558–62. 10482155 10.1016/s0002-9149(99)00377-x

[17] Kannel WB. Factors of risk in the development of coronary heart disease—six-year follow-up experience. Ann Intern Med. (1961) 55:33. 10.7326/0003-4819-55-1-33 13751193

[18] Levy D Garrison R Savage D Kannel W Castelli W. Prognostic implications of echocardiographically determined left ventricular mass in the Framingham Heart Study. N Engl J Med. (1990) 322:1561–6. 10.1056/nejm199005313222203 2139921

[19] Tsao CW Gona PN Salton CJ Chuang ML Levy D Manning WJ. Left ventricular structure and risk of cardiovascular events: a framingham heart study cardiac magnetic resonance study. J Am Heart Assoc. (2015) 4:e002188. 10.1161/JAHA.115.002188 26374295 PMC4599505

[20] Bluemke DA Kronmal RA Lima JAC Liu K Olson J Burke GL. The relationship of left ventricular mass and geometry to incident cardiovascular events. The MESA (Multi-Ethnic Study of Atherosclerosis) Study. J Am Coll Cardiol. (2008) 52:2148–55. 10.1016/j.jacc.2008.09.014 19095132 PMC2706368

[21] Rathi VK Doyle M Yamrozik J Williams RB Caruppannan K Truman C. Routine evaluation of left ventricular diastolic function by cardiovascular magnetic resonance: a practical approach. J Cardiovasc Magn Reson. (2008) 10:36. 10.1186/1532-429X-10-36 18611254 PMC2481245

[22] Bollache E Redheuil A Clément-Guinaudeau S Defrance C Perdrix L Ladouceur M. Automated left ventricular diastolic function evaluation from phase-contrast cardiovascular magnetic resonance and comparison with Doppler echocardiography. J Cardiovasc Magn Reson. (2010) 12:1–11. 10.1186/1532-429X-12-63 21062448 PMC2991299

[23] Buss SJ Krautz B Schnackenburg B Abdel-Aty H Santos MFB Andre F. Classification of diastolic function with phase-contrast cardiac magnetic resonance imaging: validation with echocardiography and age-related reference values. Clin Res Cardiol. (2014) 103:441–50. 10.1007/s00392-014-0669-3 24452509

[24] Lin E Alessio A. What are the basic concepts of temporal, contrast, and spatial resolution in cardiac CT? J Cardiovasc Comput Tomogr. (2009) 3:403. 10.1016/J.JCCT.2009.07.003 19717355 PMC4752333

[25] Chamsi-Pasha MA Zhan Y Debs D Shah DJ. CMR in the evaluation of diastolic dysfunction and phenotyping of HFpEF: current role and future perspectives. JACC Cardiovasc Imaging. (2020) 13:283–96. 10.1016/j.jcmg.2019.02.031 31202753

[26] Gulsin GS Singh A McCann GP. Cardiovascular magnetic resonance in the evaluation of heart valve disease. BMC Med Imaging. (2017) 17:1–14. 10.1186/S12880-017-0238-0/FIGURES/16 29284450 PMC5747097

[27] Fyrdahl A Ramos JG Eriksson MJ Caidahl K Ugander M Sigfridsson A. Sector-wise golden-angle phase contrast with high temporal resolution for evaluation of left ventricular diastolic dysfunction. Magn Reson Med. (2020) 83:1310–21. 10.1002/MRM.28018 31631403 PMC6972568

[28] Brandts A Bertini M Van Dijk EJ Delgado V Marsan NA Van Der Geest RJ. Left ventricular diastolic function assessment from three-dimensional three-directional velocity-encoded MRI with retrospective valve tracking. J Magn Reson Imaging. (2011) 33:312–9. 10.1002/jmri.22424 21274972

[29] Crandon S Westenberg JJM Swoboda PP Fent GJ Foley JRJ Chew PG. Impact of age and diastolic function on novel, 4D flow CMR biomarkers of left ventricular blood flow kinetic energy. Sci Rep. (2018) 8:1–11. 10.1038/s41598-018-32707-5 30258186 PMC6158175

[30] Ho CY Solomon SD. A clinician's guide to tissue doppler imaging. Circulation (2006) 113:579268. 10.1161/CIRCULATIONAHA.105.579268 16534017

[31] Nagueh SF Smiseth OA Appleton CP Byrd BF Dokainish H Edvardsen T. Recommendations for the evaluation of left ventricular diastolic function by echocardiography: an update from the american society of echocardiography and the European Association of Cardiovascular Imaging. Eur Hear J Cardiovasc Imaging. (2016) 17:1321–60. 10.1093/ehjci/jew082 27422899

[32] Paelinck BP De Roos A Bax JJ Bosmans JM Van Der Geest RJ Dhondt D. Feasibility of tissue magnetic resonance imaging: a pilot study in comparison with tissue Doppler imaging and invasive measurement. J Am Coll Cardiol. (2005) 45:1109–16. 10.1016/j.jacc.2004.12.051 15808772

[33] Okayama S Nakano T Uemura S Fujimoto S Somekawa S Watanabe M. Evaluation of left ventricular diastolic function by fractional area change using cine cardiovascular magnetic resonance: a feasibility study. J Cardiovasc Magn Reson. (2013) 15:2–7. 10.1186/1532-429X-15-87 24070403 PMC3815234

[34] Marwick TH. Ejection fraction pros and cons: JACC state-of-the-art review. J Am Coll Cardiol. (2018) 72:2360–79. 10.1016/J.JACC.2018.08.2162 30384893

[35] Potter E Marwick TH. Assessment of left ventricular function by echocardiography: the case for routinely adding global longitudinal strain to ejection fraction. JACC Cardiovasc Imaging. (2018) 11:260–74. 10.1016/J.JCMG.2017.11.017 29413646

[36] Cikes M Solomon SD. Beyond ejection fraction: an integrative approach for assessment of cardiac structure and function in heart failure. Eur Heart J. (2016) 37:1642–50. 10.1093/EURHEARTJ/EHV510 26417058

[37] Nesbitt GC Mankad S Oh JK. Strain imaging in echocardiography: methods and clinical applications. Int J Cardiovasc Imaging. (2009) 251:9–22. 10.1007/S10554-008-9414-1 19145475

[38] Kalam K Otahal P Marwick TH. Prognostic implications of global LV dysfunction: A systematic review and meta-analysis of global longitudinal strain and ejection fraction. Heart. (2014) 100:1673–80. 10.1136/heartjnl-2014-305538 24860005

[39] Pedrizzetti G Claus P Kilner PJ Nagel E. Principles of cardiovascular magnetic resonance feature tracking and echocardiographic speckle tracking for informed clinical use. J Cardiovasc Magn Reson. (2016) 18:1–12. 10.1186/s12968-016-0269-7 27561421 PMC5000424

[40] Ambale-Venkatesh B Armstrong AC Liu CY Donekal S Yoneyama K Wu CO. Diastolic function assessed from tagged MRI predicts heart failure and atrial fibrillation over an 8-year follow-up period: the multi-ethnic study of atherosclerosis. Eur Heart J Cardiovasc Imaging. (2014) 15:442–9. 10.1093/ehjci/jet189 24145457 PMC3976111

[41] Neizel M Lossnitzer D Korosoglou G Schäufele T Lewien A Steen H. Strain-encoded (SENC) magnetic resonance imaging to evaluate regional heterogeneity of myocardial strain in healthy volunteers: comparison with conventional tagging. J Magn Reson Imaging. (2009) 29:99–105. 10.1002/JMRI.21612 19097105

[42] Korosoglou G Giusca S Hofmann NP Patel AR Lapinskas T Pieske B. Strain-encoded magnetic resonance: a method for the assessment of myocardial deformation. ESC Hear Fail. (2019) 6:584–602. 10.1002/EHF2.12442 31021534 PMC6676282

[43] Lapinskas T Zieschang V Erley J Stoiber L Schnackenburg B Stehning C. Strain-encoded cardiac magnetic resonance imaging: a new approach for fast estimation of left ventricular function. BMC Cardiovasc Disord. (2019) 19:1–7. 10.1186/S12872-019-1031-5/FIGURES/4 30836942 PMC6402124

[44] Pan L Stuber M Kraitchman DL Fritzges DL Gilson WD Osman NF. Real-time imaging of regional myocardial function using fast-SENC. Magn Reson Med. (2006) 55:386–95. 10.1002/MRM.20770 16402379

[45] Petersen SE Jung BA Wiesmann F Selvanayagam JB Francis JM Hennig J. Myocardial tissue phase mapping with cine phase-contrast MR imaging: regional wall motion analysis in healthy volunteers. Radiology. (2006) 238:816–26. 10.1148/radiol.2383041992 16424246

[46] Kinno M Nagpal P Horgan S Waller AH. Comparison of echocardiography, cardiac magnetic resonance, and computed tomographic imaging for the evaluation of left ventricular myocardial function: part 2 (diastolic and regional assessment). Curr Cardiol Rep. (2017) 19:1–13. 10.1007/s11886-017-0816-3 28116679

[47] Onishi T Saha SK Delgado-Montero A Ludwig DR Onishi T Schelbert EB. Global longitudinal strain and global circumferential strain by speckle-tracking echocardiography and feature-tracking cardiac magnetic resonance imaging: comparison with left ventricular ejection fraction. J Am Soc Echocardiogr. (2015) 28:587–96. 10.1016/j.echo.2014.11.018 25577185

[48] Kuetting D Sprinkart AM Doerner J Schild H Thomas D. Comparison of magnetic resonance feature tracking with harmonic phase imaging analysis (CSPAMM) for assessment of global and regional diastolic function. Eur J Radiol. (2015) 84:100–7. 10.1016/j.ejrad.2014.10.011 25467225

[49] Moody WE Taylor RJ Edwards NC Chue CD Umar F Taylor TJ. Comparison of magnetic resonance feature tracking for systolic and diastolic strain and strain rate calculation with spatial modulation of magnetization imaging analysis. J Magn Reson Imaging. (2015) 41:1000–12. 10.1002/jmri.24623 24677420

[50] Ito H Ishida M Makino W Goto Y Ichikawa Y Kitagawa K. Cardiovascular magnetic resonance feature tracking for characterization of patients with heart failure with preserved ejection fraction: correlation of global longitudinal strain with invasive diastolic functional indices. J Cardiovasc Magn Reson. (2020) 22:1–11. 10.1186/s12968-020-00636-w 32498688 PMC7271439

[51] Romano S Judd RM Kim RJ Kim HW Klem I Heitner JF. Feature-tracking global longitudinal strain predicts death in a multicenter population of patients with ischemic and nonischemic dilated cardiomyopathy incremental to ejection fraction and late gadolinium enhancement. JACC Cardiovasc Imaging. (2018) 11:1419–29. 10.1016/j.jcmg.2017.10.024 29361479 PMC6043421

[52] Kammerlander AA Kraiger JA Nitsche C Donà C Duca F Zotter-Tufaro C. Global longitudinal strain by CMR feature tracking is associated with outcome in HFPEF. JACC Cardiovasc Imaging. (2019) 12:1585–7. 10.1016/j.jcmg.2019.02.016 31005535

[53] Kowallick JT Lamata P Hussain ST Kutty S Steinmetz M Sohns JM. Quantification of left ventricular torsion and diastolic recoil using cardiovascular magnetic resonance myocardial feature tracking. PLoS ONE (2014) 9:e0109164. 10.1371/journal.pone.0109164 25285656 PMC4186780

[54] Nucifora G Muser D Morocutti G Piccoli G Zanuttini D Gianfagna P. Disease-specific differences of left ventricular rotational mechanics between cardiac amyloidosis and hypertrophic cardiomyopathy. Am J Physiol Heart Circ Physiol. (2014) 307:H680–8. 10.1152/AJPHEART.00251.2014 24993044

[55] Pritchett AM Mahoney DW Jacobsen SJ Rodeheffer RJ Karon BL Redfield MM. Diastolic dysfunction and left atrial volume: a population-based study. J Am Coll Cardiol. (2005) 45:87–92. 10.1016/J.JACC.2004.09.054 15629380

[56] Tsang TSM Abhayaratna WP Barnes ME Miyasaka Y Gersh BJ Bailey KR. Prediction of cardiovascular outcomes with left atrial size: is volume superior to area or diameter? J Am Coll Cardiol. (2006) 47:1018–23. 10.1016/J.JACC.2005.08.077 16516087

[57] Souto Nacif M Dias Barranhas A Türkbey E Marchiori E Kawel N Mello RAF. Left atrial volume quantification using cardiac MRI in atrial fibrillation: Comparison of the Simpson's method with biplane area-length, ellipse, and three-dimensional methods. Diagnostic Interv Radiol. (2013) 19:213–20. 10.5152/dir.2012.002 23233400

[58] Kawel-Boehm N Hetzel SJ Ambale-Venkatesh B Captur G Francois CJ Jerosch-Herold M. Reference ranges (“normal values”) for cardiovascular magnetic resonance (CMR) in adults and children: 2020 update. J Cardiovasc Magn Reson. (2020) 22:1–63. 10.1186/S12968-020-00683-3 33308262 PMC7734766

[59] Khan MA Yang EY Zhan Y Judd RM Chan W Nabi F. Association of left atrial volume index and all-cause mortality in patients referred for routine cardiovascular magnetic resonance: a multicenter study 11 Medical and Health Sciences 1102 Cardiorespiratory Medicine and Haematology. J Cardiovasc Magn Reson. (2019) 21:1–12. 10.1186/S12968-018-0517-0/FIGURES/5 30612579 PMC6322235

[60] Schuster A Hor KN Kowallick JT Beerbaum P Kutty S. Cardiovascular magnetic resonance myocardial feature tracking: concepts and clinical applications. Circ Cardiovasc Imaging. (2016) 9:1–9. 10.1161/CIRCIMAGING.115.004077 27009468

[61] Kanagala P Arnold JR Singh A Khan JN Gulsin GS Gupta P. Intra-study and inter-technique validation of cardiovascular magnetic resonance imaging derived left atrial ejection fraction as a prognostic biomarker in heart failure with preserved ejection fraction. Int J Cardiovasc Imaging. (2020) 36:921–8. 10.1007/S10554-020-01785-W/FIGURES/3 32030576 PMC7174265

[62] Posina K McLaughlin J Rhee P Li L Cheng J Schapiro W. Relationship of phasic left atrial volume and emptying function to left ventricular filling pressure: a cardiovascular magnetic resonance study. J Cardiovasc Magn Reson. (2013) 15:1–8. 10.1186/1532-429X-15-99 24168103 PMC3874752

[63] Nguyen J Weber J Hsu B Mulyala RR Wang L Cao JJ. Comparing left atrial indices by CMR in association with left ventricular diastolic dysfunction and adverse clinical outcomes. Sci Rep. (2021) 11:1–10. 10.1038/s41598-021-00596-w 34716361 PMC8556227

[64] Gupta S Matulevicius SA Ayers CR Berry JD Patel PC Markham DW. Left atrial structure and function and clinical outcomes in the general population. Eur Heart J. (2013) 34:278–85. 10.1093/EURHEARTJ/EHS188 22782941 PMC3549524

[65] Markman TM Habibi M Venkatesh BA Zareian M Wu C Heckbert SR. Association of left atrial structure and function and incident cardiovascular disease in patients with diabetes mellitus : results from multi-ethnic study of atherosclerosis (MESA). Eur Heart J. (2017) 18:1138–44. 10.1093/ehjci/jew332 28329137 PMC5837690

[66] Kanagala P Arnold JR Cheng ASH Singh A Khan JN Gulsin GS. Left atrial ejection fraction and outcomes in heart failure with preserved ejection fraction. Int J Cardiovasc Imaging. (2019) 36:101–10. 10.1007/s10554-019-01684-9 31401742 PMC6942575

[67] Kowallick JT Kutty S Edelmann F Chiribiri A Villa A Steinmetz M. Quantification of left atrial strain and strain rate using Cardiovascular Magnetic Resonance myocardial feature tracking: a feasibility study. J Cardiovasc Magn Reson. (2014) 16:1–9. 10.1186/s12968-014-0060-6 25196447 PMC4422260

[68] Chirinos JA Sardana M Ansari B Satija V Kuriakose D Edelstein I. Left atrial phasic function by cardiac magnetic resonance feature tracking is a strong predictor of incident cardiovascular events. Circ Cardiovasc Imaging. (2018) 11:e007512. 10.1161/CIRCIMAGING.117.007512 30562112 PMC6301081

[69] Habibi M Chahal H Opdahl A Gjesdal O Helle-Valle TM Heckbert SR. Association of CMR-measured LA function with heart failure development: results from the MESA study. JACC Cardiovasc Imaging. (2014) 7:570–9. 10.1016/j.jcmg.2014.01.016 24813967 PMC4129378

[70] Von Roeder M Rommel KP Kowallick JT Blazek S Besler C Fengler K. Influence of left atrial function on exercise capacity and left ventricular function in patients with heart failure and preserved ejection fraction. Circ Cardiovasc Imaging. (2017) 10:e005467. 10.1161/CIRCIMAGING.116.005467 28360259

[71] Kanagala P Arnold JR Singh A Khan JN Gulsin GS Gupta P. Prevalence of right ventricular dysfunction and prognostic significance in heart failure with preserved ejection fraction. Int J Cardiovasc Imaging. (2021) 37:255–66. 10.1007/S10554-020-01953-Y/TABLES/5 32737707 PMC7878207

[72] Hur DJ Sugeng L. Non-invasive multimodality cardiovascular imaging of the right heart and pulmonary circulation in pulmonary hypertension. Front Cardiovasc Med. (2019) 6:24. 10.3389/FCVM.2019.00024/BIBTEX 30931315 PMC6427926

[73] Borlaug BA Paulus WJ. Heart failure with preserved ejection fraction: pathophysiology, diagnosis, and treatment. Eur Heart J. (2011) 32:670–9. 10.1093/eurheartj/ehq426 21138935 PMC3056204

[74] Neilan TG Shah R V Abbasi SA Farhad H Groarke JD Dodson JA. The incidence, pattern, and prognostic value of left ventricular myocardial scar by late gadolinium enhancement in patients with atrial fibrillation. J Am Coll Cardiol. (2013) 62:2205–14. 10.1016/J.JACC.2013.07.067 23994399 PMC3908872

[75] Kwong RY Sattar H Wu H Vorobiof G Gandla V Steel K. Incidence and prognostic implication of unrecognized myocardial scar characterized by cardiac magnetic resonance in diabetic patients without clinical evidence of myocardial infarction. Circulation. (2008) 118:1011–20. 10.1161/CIRCULATIONAHA.107.727826 18725488 PMC2743310

[76] Wong TC Piehler KM Zareba KM Lin K Phrampus A Patel A. Myocardial damage detected by late gadolinium enhancement cardiovascular magnetic resonance is associated with subsequent hospitalization for heart failure. J Am Heart Assoc. (2013) 2:e000416. 10.1161/JAHA.113.000416 24249712 PMC3886781

[77] Azadani PN King JB Kheirkhahan M Chang L Marrouche NF Wilson BD. Left atrial fibrosis is associated with new-onset heart failure in patients with atrial fibrillation. Int J Cardiol. (2017) 248:161–5. 10.1016/j.ijcard.2017.07.007 28735758

[78] Duca F Kammerlander AA Zotter-Tufaro C Aschauer S Schwaiger ML Marzluf BA. Interstitial fibrosis, functional status, and outcomes in heart failure with preserved ejection fraction. Circ Cardiovasc Imaging. (2016) 9:e005277. 10.1161/CIRCIMAGING.116.005277/-/DC1 27974408

[79] Messroghli DR Moon JC Ferreira VM Grosse-Wortmann L He T Kellman P. Clinical recommendations for cardiovascular magnetic resonance mapping of T1, T2, T2^*^ and extracellular volume: a consensus statement by the Society for Cardiovascular Magnetic Resonance (SCMR) endorsed by the European Association for Cardiovascular Imaging (EACVI). J Cardiovasc Magn Reson. (2017) 19:1–24. 10.1186/S12968-017-0389-8 28992817 PMC5633041

[80] Su MYM Lin LY Tseng YHE Chang CC Wu CK Lin JL. CMR-verified diffuse myocardial fibrosis is associated with diastolic dysfunction in HFpEF. JACC Cardiovasc Imaging. (2014) 7:991–7. 10.1016/J.JCMG.2014.04.022 25240451

[81] Rommel KP Von Roeder M Latuscynski K Oberueck C Blazek S Fengler K. Extracellular volume fraction for characterization of patients with heart failure and preserved ejection fraction. J Am Coll Cardiol. (2016) 67:1815–25. 10.1016/J.JACC.2016.02.018 27081022

[82] Omori T Nakamori S Fujimoto N Ishida M Kitagawa K Ichikawa Y. Myocardial native T 1 predicts load-independent left ventricular chamber stiffness in patients with HFpEF. JACC Cardiovasc Imaging. (2020) 13:2117–28. 10.1016/J.JCMG.2020.05.030 32771571

[83] Mordi IR Singh S Rudd A Srinivasan J Frenneaux M Tzemos N. Comprehensive echocardiographic and cardiac magnetic resonance evaluation differentiates among heart failure with preserved ejection fraction patients, hypertensive patients, and healthy control subjects. JACC Cardiovasc Imaging. (2018) 11:577–85. 10.1016/J.JCMG.2017.05.022 28823736

[84] Roy C Slimani A De Meester C Amzulescu M Pasquet A Vancraeynest D. Associations and prognostic significance of diffuse myocardial fibrosis by cardiovascular magnetic resonance in heart failure with preserved ejection fraction. J Cardiovasc Magn Reson. (2018) 20:1–12. 10.1186/S12968-018-0477-4 30086783 PMC6081897

[85] Schelbert EB Fridman Y Wong TC Abu Daya H Piehler KM Kadakkal A. Temporal relation between myocardial fibrosis and heart failure with preserved ejection fraction: association with baseline disease severity and subsequent outcome. JAMA Cardiol. (2017) 2:995–1006. 10.1001/JAMACARDIO.2017.2511 28768311 PMC5710176

[86] Mascherbauer J Marzluf BA Tufaro C Pfaffenberger S Graf A Wexberg P. Cardiac magnetic resonance postcontrast T1 time is associated with outcome in patients with heart failure and preserved ejection fraction. Circ Cardiovasc Imaging. (2013) 6:1056–65. 10.1161/CIRCIMAGING.113.000633 24036385

[87] Kanagala P Cheng ASH Singh A Khan JN Gulsin GS Patel P. Relationship between focal and diffuse fibrosis assessed by CMR and clinical outcomes in heart failure with preserved ejection fraction. JACC Cardiovasc Imaging. (2019) 12:2291–301. 10.1016/J.JCMG.2018.11.031 30772227

[88] Nitsche C Kammerlander AA Binder C Duca F Aschauer S Koschutnik M. Native T1 time of right ventricular insertion points by cardiac magnetic resonance: relation with invasive haemodynamics and outcome in heart failure with preserved ejection fraction. Eur Heart J Cardiovasc Imaging. (2020) 21:683–91. 10.1093/EHJCI/JEZ221 31495874

[89] Guazzi M Adams V Conraads V Halle M Mezzani A Vanhees L. Clinical recommendations for cardiopulmonary exercise testing data assessment in specific patient populations. Circulation. (2012) 126:2261–74. 10.1161/CIR.0b013e31826fb946 22952317 PMC4777325

[90] Obokata M Kane GC Reddy YNV Olson TP Melenovsky V Borlaug BA. Role of diastolic stress testing in the evaluation for heart failure with preserved ejection fraction: a simultaneous invasive-echocardiographic study. Circulation. (2017) 135:825–38. 10.1161/CIRCULATIONAHA.116.024822 28039229 PMC5330848

[91] Kato S Nakamori S Roujol S Delling FN Akhtari S Jang J. Relationship between native papillary muscle T1 time and severity of functional mitral regurgitation in patients with non-ischemic dilated cardiomyopathy. J Cardiovasc Magn Reson. (2017) 18:79. 10.1186/s12968-016-0301-y 27846845 PMC5111188

[92] Rush CJ Berry C Oldroyd KG Rocchiccioli JP Lindsay MM Touyz RM. Prevalence of coronary artery disease and coronary microvascular dysfunction in patients with heart failure with preserved ejection fraction. JAMA Cardiol. (2021) 6:1130–43. 10.1001/JAMACARDIO.2021.1825 34160566 PMC8223134

[93] Backhaus SJ Lange T George EF Hellenkamp K Gertz RJ Billing M. Exercise stress real-time cardiac magnetic resonance imaging for noninvasive characterization of heart failure with preserved ejection fraction: the HFpEF-stress trial. Circulation (2021) 143:1484–98. 10.1161/CIRCULATIONAHA.120.051542 33472397

[94] Löffler AI Pan JA Balfour PC Shaw PW Yang Y Nasir M. Coronary microvascular dysfunction and diffuse myocardial fibrosis measured by cardiovascular magnetic resonance are characteristic of HFpEF. Am J Cardiol. (2019) 124:1584. 10.1016/J.AMJCARD.2019.08.011 31575425 PMC6894613

[95] Pezel T Hovasse T Sanguineti F Kinnel M Garot P Champagne S. Long-term prognostic value of stress CMR in patients with heart failure and preserved ejection fraction. Cardiovasc Imaging. (2021) 14:2319–33. 10.1016/J.JCMG.2021.03.010 34419409

[96] Arnold JR Kanagala P Budgeon CA Jerosch-Herold M Gulsin GS Singh A. Prevalence and prognostic significance of microvascular dysfunction in heart failure with preserved ejection fraction. JACC Cardiovasc Imaging (2022) 15:1001–11. 10.1016/J.JCMG.2021.11.022 35033490

[97] Diamant M Lamb HJ Groeneveld Y Endert EL Smit JWA Bax JJ. Diastolic dysfunction is associated with altered myocardial metabolism in asymptomatic normotensive patients with well-controlled type 2 diabetes mellitus. J Am Coll Cardiol. (2003) 42:328–35. 10.1016/s0735-1097(03)00625-9 12875772

[98] Burrage MK Hundertmark M Valkovič L Watson WD Rayner J Sabharwal N. Energetic basis for exercise-induced pulmonary congestion in heart failure with preserved ejection fraction. Circulation. (2021) 144:1664–78. 10.1161/circulationaha.121.054858 34743560 PMC8601674

[99] Mahmod M Pal N Rayner J Holloway C Raman B Dass S. The interplay between metabolic alterations, diastolic strain rate and exercise capacity in mild heart failure with preserved ejection fraction: A cardiovascular magnetic resonance study. J Cardiovasc Magn Reson. (2018) 20:1–10. 10.1186/S12968-018-0511-6/TABLES/3 30580760 PMC6304764

[100] Anker SD Butler J Filippatos G Ferreira JP Bocchi E Böhm M. Empagliflozin in heart failure with a preserved ejection fraction. N Engl J Med. (2021) 385:1451–61. 10.1056/NEJMOA2107038/SUPPL_FILE/NEJMOA2107038_DATA-SHARING.PDF 34449189

[101] Thirunavukarasu S Jex N Chowdhary A Hassan IU Straw S Craven TP. Empagliflozin treatment is associated with improvements in cardiac energetics and function and reductions in myocardial cellular volume in patients with type 2 diabetes. Diabetes. (2021) 70:2810–22. 10.2337/DB21-0270 34610982 PMC8660983

[102] Rao VN Fudim M Mentz RJ Michos ED Felker GM. Regional adiposity and heart failure with preserved ejection fraction. Eur J Heart Fail. (2020) 22:1540–50. 10.1002/EJHF.1956 32619081 PMC9991865

[103] Obokata M Reddy YNV Pislaru S V Melenovsky V Borlaug BA. Evidence supporting the existence of a distinct obese phenotype of heart failure with preserved ejection fraction. Circulation. (2017) 136:6–19. 10.1161/CIRCULATIONAHA.116.026807/-/DC1 28381470 PMC5501170

[104] Mendoza D Codella N Wang Y Prince M Sethi S Manoushagian S. Impact of diastolic dysfunction severity on global left ventricular volumetric filling—assessment by automated segmentation of routine cine cardiovascular magnetic resonance. J Cardiovasc Magn Reson. (2010) 12:1–11. 10.1186/1532-429X-12-46 20673372 PMC2924850

[105] Gao C Tao Y Pan J Shen C Zhang J Xia Z. Evaluation of elevated left ventricular end diastolic pressure in patients with preserved ejection fraction using cardiac magnetic resonance. Eur Radiol. (2019) 29:2360–8. 10.1007/s00330-018-5955-4 30631923

[106] Hieda M Parker J Rajabi T Fujimoto N Bhella PS Prasad A. Left ventricular volume-time relation in patients with heart failure with preserved ejection fraction. Am J Cardiol. (2018) 121:609–14. 10.1016/j.amjcard.2017.11.033 29306483 PMC6545885

[107] Kawaji K Codella NCF Prince MR Chu CW Shakoor A LaBounty TM. Automated segmentation of routine clinical cardiac magnetic resonance imaging for assessment of left ventricular diastolic dysfunction. Circ Cardiovasc Imaging. (2009) 2:476–84. 10.1161/CIRCIMAGING.109.879304 19920046

[108] Reiter G Reiter U Kovacs G Kainz B Schmidt K Maier R. Magnetic resonance-derived 3-dimensional blood flow patterns in the main pulmonary artery as a marker of pulmonary hypertension and a measure of elevated mean pulmonary arterial pressure. Circ Cardiovasc Imaging. (2008) 1:23–30. 10.1161/CIRCIMAGING.108.780247 19808511

[109] Reiter U Reiter G Kovacs G Stalder AF Gulsun MA Greiser A. Evaluation of elevated mean pulmonary arterial pressure based on magnetic resonance 4D velocity mapping: comparison of visualization techniques. PLoS ONE. (2013) 8:e82212. 10.1371/JOURNAL.PONE.0082212 24349224 PMC3861394

[110] Reiter G Reiter U Kovacs G Olschewski H Fuchsjäger M. Blood flow vortices along the main pulmonary artery measured with MR imaging for diagnosis of pulmonary hypertension. Radiology. (2015) 275:71–9. 10.1148/RADIOL.14140849 25372980

[111] Ramos JG Fyrdahl A Wieslander B Reiter G Reiter U Jin N. Cardiovascular magnetic resonance 4D flow analysis has a higher diagnostic yield than Doppler echocardiography for detecting increased pulmonary artery pressure. BMC Med Genet. (2020) 21:1–9. 10.1186/S12880-020-00428-9 32143594 PMC7060590

[112] Shors SM Cotts WG Pavlovic-Surjancev B François CJ Gheorghiade M Finn JP. Heart failure: evaluation of cardiopulmonary transit times with time-resolved. MR Angiography. (2003) 229:743–8. 10.1148/RADIOL.2293021363 14657311

[113] Cao JJ Li L McLaughlin J Passick M. Prolonged central circulation transit time in patients with HFpEF and HFrEF by magnetic resonance imaging. Eur Heart J Cardiovasc Imaging. (2018) 19:339–46. 10.1093/ehjci/jex051 28387860

[114] Khalique Z Ferreira PF Scott AD Nielles-Vallespin S Firmin DN Pennell DJ. Diffusion tensor cardiovascular magnetic resonance imaging: a clinical perspective. JACC Cardiovasc Imaging. (2020) 13:1235–55. 10.1016/J.JCMG.2019.07.016 31607663

[115] Arani A Arunachalam SP Chang ICY Baffour F Rossman PJ Glaser KJ. Cardiac MR elastography for quantitative assessment of elevated myocardial stiffness in cardiac amyloidosis. J Magn Reson Imaging. (2017) 46:1361–7. 10.1002/JMRI.25678 28236336 PMC5572539

[116] Raisi-Estabragh Z Izquierdo C Campello VM Martin-Isla C Jaggi A Harvey NC. Cardiac magnetic resonance radiomics: basic principles and clinical perspectives. Eur Hear J - Cardiovasc Imaging. (2020) 21:349–56. 10.1093/EHJCI/JEAA028 32142107 PMC7082724

